# Effects of Freezing *Lycorma delicatula* Egg Masses on Nymph Emergence and Parasitization by *Anastatus orientalis*

**DOI:** 10.3389/finsc.2022.937129

**Published:** 2022-07-12

**Authors:** Francesc Gómez-Marco, Mark S. Hoddle

**Affiliations:** ^1^ Department of Entomology, University of California Riverside, Riverside, CA, United States; ^2^ Center for Invasive Species Research, University of California Riverside, Riverside, CA, United States

**Keywords:** biotic resistance, Eupelmidae, Fulgoridae, invasive species, native parasitoids, proactive biological control, sentinel eggs

## Abstract

*Lycorma delicatula* (White) (Hemiptera: Fulgoridae), native to China, was first detected in Pennsylvania, U.S. in 2014. This polyphagous pest can feed on over 70 plant species including agricultural crops, like grapes, that have high economic value. *Anastatus orientalis* Yang and Choi (Hymenoptera: Eupelmidae) is an egg parasitoid associated with *L. delicatula* egg masses in China that is being evaluated for possible introduction into the U.S. for classical biological control of *L. delicatula*. In support of this program, the suitability of frozen *L. delicatula* eggs for parasitization by *A. orientalis* was evaluated in a quarantine laboratory. Host egg masses held for four different cold storage periods (5°C for <1, 4, 8 and 11 months) were frozen at -40°C for 1 hour or 24 hours and exposed to female *A. orientalis* for parasitization for seven days. Following this experimental exposure period, rates of *L. delicatula* nymph emergence and *A. orientalis* parasitism were assessed for each of the eight different cold storage treatments. Host acceptance and suitability of frozen *L. delicatula* eggs by *A. orientalis* was assessed in terms of percentage parasitism, offspring sex ratio, and hind tibia length of emerged parasitoids. Results indicated that *L. delicatula* nymphs failed to emerge from eggs that were exposed to -40°C for 1 hour and 24 hours and *A. orientalis* could successfully parasitize *L. delicatula* eggs regardless of cold storage and freezing treatment. These results add a new tool for long term maintenance of *L. delicatula* egg masses and rearing methods for egg parasitoids of this pest. Additionally, it may be possible to field deploy sentinel eggs of *L. delicatula* frozen at -40°C to survey for resident natural enemy species capable of parasitizing eggs of this pest in advance of anticipated *L. delicatula* invasions into new areas.

## Introduction

Spotted lanternfly, *Lycorma delicatula* (White) (Hemiptera: Fulgoridae), native to China (1), is an invasive species in South Korea and Japan where it invaded in 2004 and 2009, respectively (2, 3). In September 2014, *L. delicatula* was detected for the first time in Berks County, Pennsylvania, U.S. By April 2022, *L. delicatula* infestations were confirmed in an additional ten states in eastern (Connecticut, Delaware, Maryland, Massachusetts, New Jersey, New York, Virginia, and West Virginia) and mid-western (Indiana and Ohio) areas of the U.S (4). *Lycorma delicatula* is a phloem-feeding fulgorid that has a broad host range having been recorded feeding on over 70 plant species encompassing 25 families (5–7). Feeding by high density populations causes direct damage to host plants through removal of phloem fluids and indirect damage results from the excretion of high quantities of honeydew that promote sooty mold growth (5, 8). Direct feeding damage can cause mortality to highly preferred hosts *Ailanthus altissima* (Miller) (Sapindales: Simaroubaceae) and grapevines (*Vitis vinifera* L. [Vitales: Vitaceae]). *Lycorma delicatula* is also of high concern for other economically important perennial agricultural crops like fruit (e.g., apples, *Malus domestica* Borkh [Rosales: Rosaceae]) and nut (e.g., walnuts, *Juglans* spp. [Fagales: Juglandaceae]) trees (4). Additionally, *L. delicatula* has been recorded infesting forest and ornamental shade tree species (5, 9). *Lycorma delicatula* can disperse short distances through wind-assisted gliding and long-distance dispersal is almost entirely through human-assisted movement. This occurs primarily through the accidental translocation of egg masses that are often laid indiscriminately on inert substrates (e.g., wooden pallets and railcars) that undergo subsequent transportation into uninfested areas (10–12). This type of inadvertent relocation resulted in the establishment of invasion bridgeheads in the mid-west (e.g., Indiana and Ohio) and northeastern U.S. (e.g., Massachusetts) in 2021 (4). Tree of heaven, *A. altissima*, a widely distributed and common invasive plant, is a highly preferred host for *L. delicatula* and the high abundance of this tree has likely facilitated the invasion success of *L. delicatula* (5). Waki et al. (13) modeled the potential distribution of *L. delicatula* and results indicated that large areas of the west coast of the U.S., and other parts of the world (e.g., Europe), have high potential climatic suitability for *L. delicatula* establishment and proliferation. With respect to California, a western U.S. state with an agricultural economy worth ~$50 billion per year (14), *L. delicatula* is viewed as a significant invasion threat that could cause significant problems for producers of specialty crops like grapes and nuts. Consequently, *L. delicatula* is the subject of a proactive biological control research program that is being undertaken in advance of its anticipated incursion and establishment in California (15).

*Anastatus orientalis* Yang and Choi (Hymenoptera: Eupelmidae) is an egg parasitoid that was discovered parasitizing *L. delicatula* eggs in northern China in 2011. This parasitoid was found during foreign exploration surveys for natural enemies for potential use in a classical biological control program targeting invasive *L. delicatula* populations in South Korea (16, 17). Interest in the use of *A. orientalis* as a classical biological control agent increased significantly in 2015 following the invasion and spread of *L. delicatula* in the U.S (18).. Accordingly, the proactive biological control program underway in California targeting *L. delicatula* has focused research efforts on *A. orientalis*. Broadley et al. (18) investigated aspects of the biology and rearing of *A. orientalis.* One finding from this study was that the number and sex ratio of progeny produced and parasitism rates of *A. orientalis* per *L. delicatula* egg mass did not differ between newly collected eggs vs. eggs stored at 5°C for up to 10 months. However, this study did not examine the number of *L. delicatula* nymphs that emerged from freshly collected *L. delicatula* egg masses, those stored at 5°C, or what nymph emergence rates would be if eggs were stored at temperatures below 5°C (e.g., -40°C) for varying time periods. Previous studies have shown that parasitoid species in the genus *Anastatus* were able to parasitize frozen hemipteran and lepidopteran eggs (19, 20). However, it is unknown if fulgorid egg masses (e.g., *L. delicatula*) would be suitable for parasitism by *A. orientalis* after freezing.

There are two objectives for this study, first, to test if exposure to -40°C for ≤ 3 days can kill *L. delicatula* eggs. The USDA-APHIS approved protocol for killing *L. delicatula* eggs (and nymphs and adults) at the Insectary and Quarantine Facility at the University of California Riverside, is to hold life stages at -40°C for 72 hours before properly removing them from the quarantine facility (USDA-APHIS Permit to Move Live Plants Pests, Noxious Weeds, and Soil number P526P-19-02058). A lethal period shorter than 72 hours for *L. delicatula* eggs may be possible and warrants determination. Second, when this study was undertaken, it was unknown if *L. delicatula* eggs killed by freezing would be suitable for parasitism by *A. orientalis*. Answering this question could increase cold storage options for *L. delicatula* eggs for laboratory rearing of *A. orientalis*. Additionally, if eggs are successfully killed at -40°C and are acceptable for parasitization by egg parasitoids it may be possible to proactively deploy unviable sentinel egg masses to survey for resident natural enemy species capable of parasitizing *L. delicatula* egg masses in areas identified as being at risk of invasion. Surveys of this nature could provide a potential measure of naturally occurring levels of biotic resistance in advance of an anticipated incursion.

## Material and Methods

### Source of *Lycorma Delicatula* Eggs for Experiments

A total of 1,554 *L. delicatula* egg masses were field collected in February and December of 2020 of which 263 egg masses with 45.4 ± 1.26 eggs/egg mass (plus 53 egg masses for pre-oviposition purposes [see below]) were randomly selected and used for experiments reported here. Collections were made in seven different locations in Pennsylvania, U.S. from *A. altissima* (**Table 1**). Entire egg masses attached to underlying bark were removed using chisels and shipped to the University of California Riverside Insectary and Quarantine Facility (UCR-IQF) under USDA-APHIS permit P526P-19-02058 and California Department of Food and Agriculture (CDFA) Permit 3458. In quarantine, all field collected egg masses were stored at 5°C and 60-75% R.H. for < 1 month, 4, 8, or 11 months until used for experiments (see below for details).

**Table 1:** *Lycorma delicatula* egg mass collection sites and dates in Pennsylvania, U.S.

County	Location	Number of egg masses	Collection date	GPS
Berks	Reading	58	5-Feb-20	40°21’18.275’’N-75°55’38424’’W
Dauphin	Harrisburg	137	24-Feb-20	40°15’58.719’’N-76°53’10.003’’W
Huntingdon	Petersburg	171	20-Feb-20	40°34’19.95’’N-78°2’51.633’’W
Lancaster	Lancaster	50	1-Dec-20	40°2’17.268’’N-76°18’20.406’’W
Lebanon	Lebanon	837	26-Feb-20	40°22’32.567’’N-76°27’45.402’’W
	Myerstown	191	4-Feb-20	40°22’27.157’’N-76°18’13.167’’W
	Palmyra	110	18-Dec-20	40°18’18.111’’N-76°35’30.468’’W
**Total**		1,554		

The *A. orientalis* colony was established in UCR-IQF from parasitized *L. delicatula* egg masses shipped under USDA-APHIS permit P526P-19-02066 from USDA-APHIS-PPQ Science and Technology, Buzzard Bay, Massachusetts U.S. in October of 2019. The USDA-APHIS colony was initiated with parasitoids originally reared from *L. delicatula* eggs collected in Beijing, China, the source country of the invasive *L. delicatula* population in the U.S (18). Upon receipt at UCR-IQF, parasitized egg masses were held at 25°C and R.H. 65% for ~40 days for *A. orientalis* to complete emergence. Emerged parasitoids were used to initiate colonies [see Broadley et al. (18) for *A. orientalis* rearing protocols] that were maintained on field collected *L. delicatula* egg masses (**Table 1**) in UCR-IQF.

### *Lycorma delicatula* Egg Storage Periods and Freezing Treatments

In UCR-IQF, field collected *L. delicatula* egg masses were stored for four different periods: <1 month, 4, 8, and 11 months at 5°C before use in experiments. For each experimental cold storage period, <1 month, 4 months, 8 and 11 months, 57, 84, 24 and 24 egg masses, respectively, were randomly selected and subdivided to make eight experimental groups each of which was exposed to -40°C for two times intervals, 1 hour (egg masses <1 month, n = 17; 4 months, n = 39; 8 months and 11 months, n = 12 each) or 24 hours (egg masses <1 month, n = 23; 4 months, n = 45; 8 months and 11 months, n = 12 each) (**Table 2**). After both freezing treatments at -40°C, egg masses were “thawed” at room temperature (~25°C) for 30 minutes before being either exposed or not exposed to female *A. orientalis* for parasitization (**Table 2**). Additionally, four groups of *L. delicatula* egg masses (i.e., unfrozen treatment) not exposed to -40°C were set up under the same conditions as cold treated egg masses to measure the emergence rates of *L. delicatula* nymphs from eggs that had experienced one of the four experimental storage periods (i.e., egg masses stored at 5°C stored for <1 month, n = 13; 4 months, n = 19; 8 and 11 months, n = 6 each). Following the same protocol, an additional treatment (i.e., an unfrozen/parasitized treatment) was set up which exposed *A. orientalis* females to egg masses stored at 5°C for each of the four experimental cold storage periods (i.e., <1 month, n = 15 egg masses; 4 months; n = 18; 8 and 11 months, n = 6 each) to determine egg suitability for parasitism (**Table 2**).

**Table 2:** Treatment assignments for experimental *Lycorma delicatula* egg masses and the number (n) of repetitions for each treatment.

Time at -40°C	Exposed to *A. orientalis*	Name of the treatment	Cold storage periods at 5°C (months)			
			<1	4	8	11
1 hour	no	1h.-40°C	n = 10	n = 15	n = 6	n = 6
	yes	1h.-40°C.Parasitism	n = 7	n = 24	n = 6	n = 6
24 hours	no	24h.-40°C	n = 12	n = 20	n = 6	n = 6
	yes	24h.-40°C.Parasitism	n = 11	n = 25	n = 6	n = 6
No exposure	no	Control	n = 13	n = 19	n = 6	n = 6
	yes	Parasitism	n = 15	n = 20	n = 6	n = 6

### Experimental Set Up and Completion of a Seven Day Pre-Oviposition Period of *A. orientalis*

All experiments were conducted in temperature and humidity controlled cabinets programmed to cycle through average Fall (i.e., September) temperatures for Beijing (average daily high 25°C, average daily low 14°C, lights on 6:00 AM, lights off 6:30 PM (i.e., L:D 12.5:11.5), 65% R.H. (see **Supplementary Table 1**) in the UCR-IQF. Fall temperatures in Beijing were used to simulate the natural conditions of the original collection area of *A. orientalis* in order to optimize parasitoid parasitism behavior and development time (18). Each experimental egg mass-parasitoid test arena was comprised of a clear plastic container 3 cm x 4 cm x 5cm (180mL clear RPTE hinged lid deli containers, AD16 GenPak, Charlotte, NC) with a modified lid that had a ventilated mesh window (1.5 cm x 2.5 cm) to facilitate air exchange. One *L. delicatula* egg mass from one of the egg storage period categories that eggs were exposed to were placed into each test unit after this seven day pre-oviposition period. The inclusion of a *L. delicatula* egg mass in this pre-oviposition period is necessary as it allows female parasitoids to mate, host feed, and mature eggs for oviposition (Gomez et al. manuscript in preparation). Five female and one male *A. orientalis*, ~24 hours of age, were introduced onto egg masses to mate and for females to complete their pre-oviposition period (18). Streaks of honey were applied to lids to provide a carbohydrate source for parasitoids and arenas were sealed with the ventilated lid. Following the seven day preoviposition period, egg masses and parasitoids were removed from test arenas. Male *A. orientalis* were replaced if they died. Females were not replaced because of the seven-day exposure required for new females (i.e., ~24 hours of age) to reach maximum parasitism performance.

### Host Emergence and Parasitism Rates, Offspring Sex Ratio

Following the seven day pre-oviposition period, parasitoids were provided either one treated (i.e., -40°C for 1 hour or 24 hours) egg mass from one of the four egg storage periods (i.e., < 1, 4, 8 or 11 months at 5°C) or non-frozen egg masses from the same four storage period categories in the experimental units described above for an additional seven days. After this seven-day period (females now had a total of 14 days exposed to *L. delicatula* egg masses), parasitoids were removed from all test units and experimental egg masses inside test arenas were placed in a temperature cabinet programmed to simulate fluctuating temperatures that parasitoids would experience during fall in Beijing China for four weeks (see above). After this four-week exposure to fluctuating temperature cycles, egg masses were held at a constant 25°C and R.H. 75% until parasitoid emergence. Data collected from experimental units included the total number of *L. delicatula* eggs per experimental egg mass, the total number of emerged *L. delicatula* nymphs per egg mass, the number and gender of emerged parasitoids and the number of unemerged parasitoids (i.e., larvae, pupae and/or adult parasitoids that died and failed to emerge from eggs were found after dissection of unhatched eggs). Percentage parasitism was calculated by dividing the number of emerged and unemerged parasitoids by the total number of *L. delicatula* eggs that comprised an egg mass which was multiplied by 100. Parasitoid sex ratio was calculated as the number of female parasitoids divided by the total number of female and male parasitoids combined that emerged from each experimental egg mass.

### Measurement of Hind Tibia Length as an Assessment of Parasitoid Fitness

To evaluate the effect of -40°C exposures for 1 hour or 24 hours treatments on *L. delicatula* egg quality for *A. orientalis* development, the fitness of female parasitoids that successfully emerged from eggs cold stored at 5°C for four months, and four month old eggs that were exposed to -40°C for 1 hour or 24 hours only were assessed by using measurements of right hind tibia lengths as a proxy for parasitoid size and subsequent fitness (i.e., parasitoids with larger hind tibia are assumed to be bigger and more fit than parasitoids with smaller tibia). The four month storage period was selected for this study since it is the average approximate length of the storage period that the *L. delicatula* egg masses would be held for prior to use in experiments. Excised right hind tibiae were placed onto glass slides and covered with a second glass slide. Hind tibia length was measured from its attachment to the femur to the attachment point with the tarsi using a Leica S8AP0 microscope. Slide mounted hind tibiae were photographed at a magnification of 25 × with an attached Leica DMC2900 camera and length was measured using the Leica Application Suite version 4.6.2. A total of 25 A*. orientalis* females from each treatment, both freezing treatments (i.e., 1 hour and 24 hours at -40°C) and the control treatment [i.e., Unfrozen/parasitized treatment (**Table 2**)] were measured for a cumulative total of 75 female tibiae.

### Statistical Analyses

All statistical analyses were performed in R 4.1.3 (21) using RStudio 2022.02.0 Build 443 (22). Untransformed data met the assumptions of selected statistical tests and models used, unless specified otherwise. To test for differences in *L. delicatula* nymph emergence and parasitism rates between cold storage times (i.e. <1, 4, 8 and 11 months at 5°C) and treatments (i.e. controls, -40°C exposure for 1 hour or 24 hours) a Kruskal-Wallis test was performed. When differences were found, these analyses were followed by multiple pairwise comparisons using Wilcoxon rank sum test in each group with Bonferroni corrections at the 0.05 level of significance.

The sex ratio of emerged *A. orientalis* females across the four different cold storage categories of egg masses (i.e., <1 month, 4, 8, and 11 months at stored 5°C) that were frozen at -40°C for either 1 hour or 24 hours and exposed to parasitoids were compared to the sex ratio of female parasitoids that emerged from control egg masses used for parasitism (i.e., <1 month, 4, 8, and 11 months at 5°C and not exposed to -40°C). These comparisons were made using a quasibinomial GLM with a logit link function that included two variables; egg storage period (i.e., the four cold storage periods, <1 month, 4, 8, and 11 months) and treatment (i.e., egg masses that were either frozen or not frozen at -40°C for 1 hour or 24 hours, and either exposed or not exposed to parasitoids). To determine if significant effects from test variables existed ANOVA was conducted followed by a Tukey posthoc test at the 0.05 level of significance to identify differences between treatment categories and cold storage exposure treatment times. Differences in mean hind tibiae lengths between parasitoids emerging from egg masses stored at 5°C for four months on the three treatment groups (i.e., -40°C for 1 or 24 hours and not frozen at -40°C) was analyzed by ANOVA followed by a Tukey posthoc test at the 0.05 level of significance. All means are presented ± SE.

## Results

### Effects of -40°C on *Lycorma delicatula* Nymph Emergence Rates

**Figure 1:**
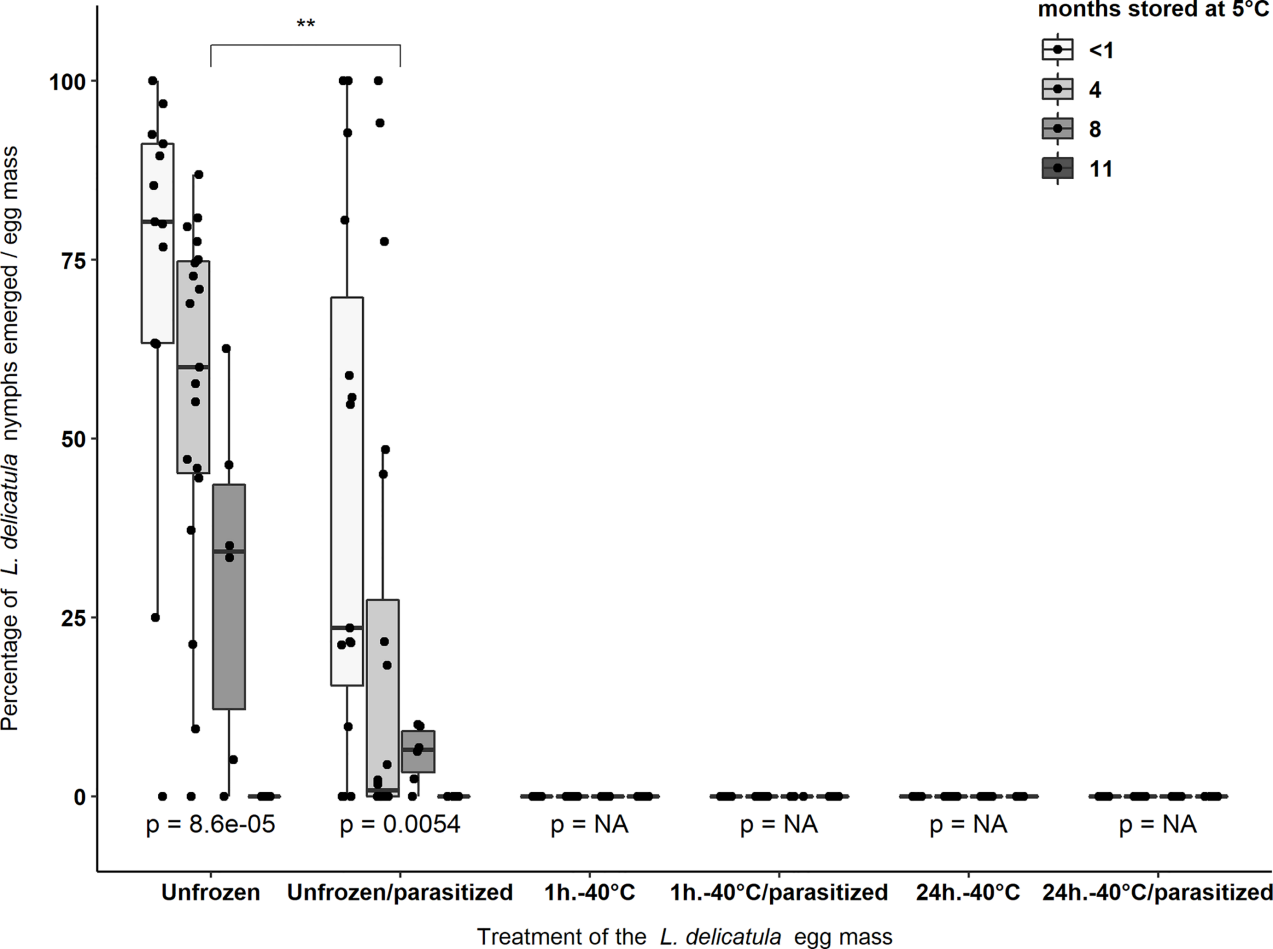
Average percentage of nymphs that emerged from *L. delicatula* egg masses stored at 5°C and 65% R.H. for four different storage periods: <1 month, 4, 8, and 11 months. After these storage periods a subset of these egg masses were frozen at -40°C for 1 hour or 24 hours and either exposed or not exposed to *A. orientalis*. Asterisks (**) indicate significant differences (*p* < 0.01) in the percentage of *L. delicatula* nymphs that emerged from egg masses between treatment groups (i.e., Unfrozen and Unfrozen/parasitized). *p*-values < 0.05 indicate significant differences between the percentage of nymphs emerged from egg masses that were stored for different periods within the same treatment group. NA “not-applicable” indicate that no statistical analyzes were used as all data were zeros in each treatment group. Black dots represent data points.

Irrespective of storage times (i.e., < 1, 4, 8, 11 months) at 5°C, when *L. delicatula* eggs were exposed to -40°C for either 1 hour or 24 hours zero nymphs emerged from a total of 7,557 eggs that comprised the 172 egg masses that were used in these two treatments (**Table 2**) (**Figure 1**).

The maximum percentage emergence of *L. delicatula* nymphs was from unfrozen egg masses not exposed to parasitoids which were stored for <1 month and four months with an average emergence of 72.6 ± 8.2% (n = 13 egg masses) and 56.1 ± 5.7% (n = 19), respectively (**Figure 1**). Around 30.4 ± 9.8% (n = 6) nymphs emerged from egg masses stored for eight months and no nymphs emerged from unfrozen egg masses that were stored for 11 months at 5°C (n = 6). Parasitism by *A. orientalis* significantly reduced the percentage of *L. delicatula* nymphs that emerged from egg masses (χ^2^ = 10.53, d.f. = 1, *p* = 0.001). This effect was significantly different in the egg masses stored for four months only (χ^2^ = 10.04, d.f. = 1, *p* = 0.001) (**Figure 1**).

For control treatments not exposed to -40°C, the extent of time (i.e., <1 month, 4, 8, and 11 months) that *L. delicatula* egg masses were stored at 5°C significantly reduced percentage nymph emergence per egg mass (unfrozen egg masses not exposed to parasitoids: χ^2^ = 21.43, d.f. = 3, *p* < 0.001; unfrozen eggs exposed to parasitoids: χ^2^ = 12.69, d.f. = 3, *p* = 0.005) (**Figure 1**).

### Parasitism of Experimental Egg Masses by *Anastatus orientalis*

**Figure 2:**
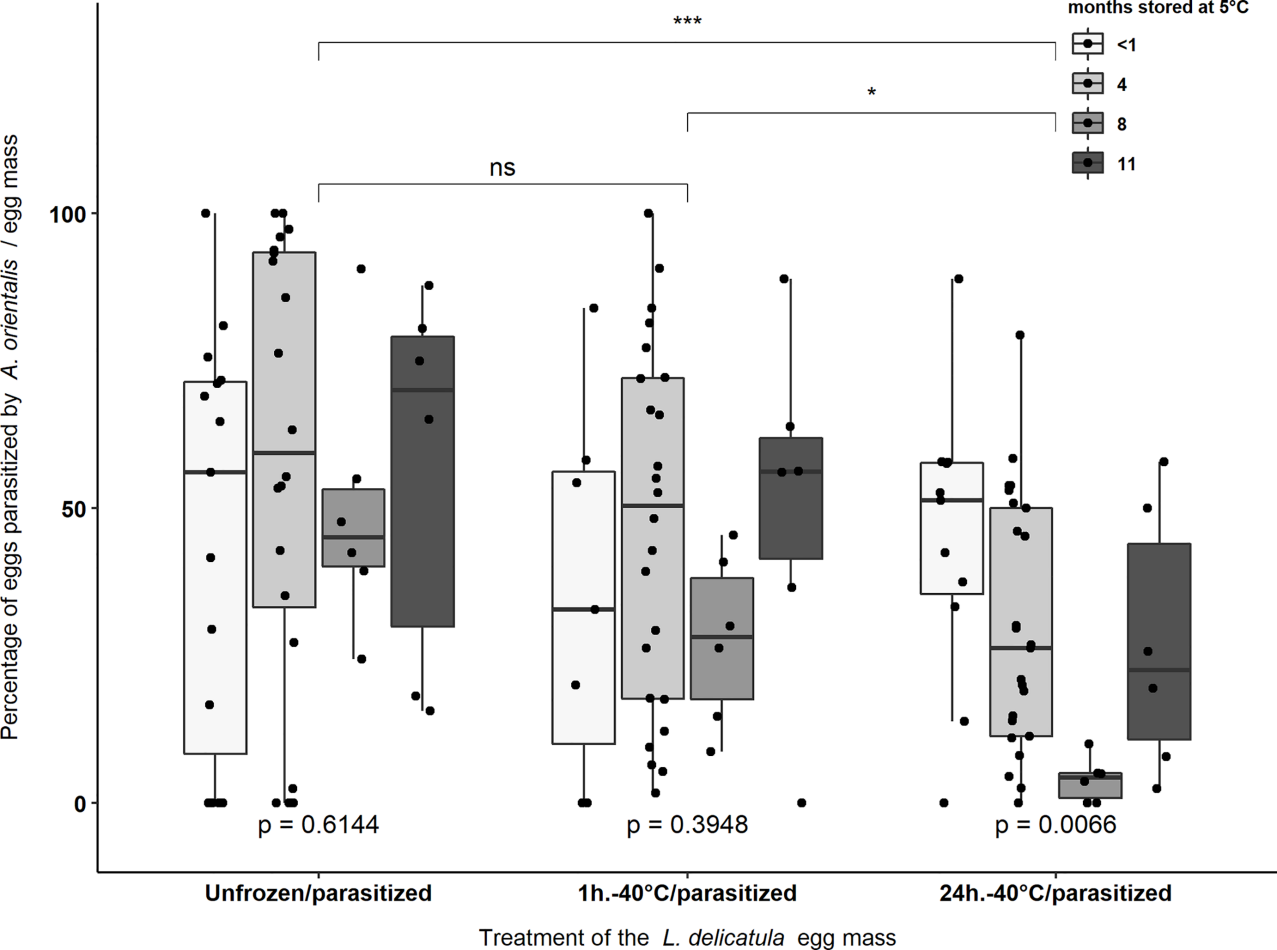
Percentage parasitism of *L. delicatula* egg masses by *A. orientalis* that were treated at -40°C for 1 hour or 24 hours or not frozen (i.e., Unfrozen/parasitized) prior to exposure to female parasitoids. Experimental egg masses were stored at 5°C and 65% R.H. at four different periods: <1 month, 4, 8, and 11 months prior to exposure to -40°C. Asterisks indicate significant differences [(*) = *p* < 0.05; (***) = *p* < 0.001] and “ns” indicate non-significant differences in the percentage of parasitism between treatment groups. p-values < 0.05 indicate significant differences in the percentage of parasitism between egg masses that were stored for different periods within the same treatment group. Black dots represent data points.

A total of 6,024 eggs from 138 *L. delicatula* egg masses (mean of 43.652 ± 1.377 eggs per egg mass) stored at 5°C for the four storage periods were provided to *A. orientalis* for parasitism following three different freezing treatments (**Table 2**). *Anastatus orientalis* was able to parasitize *L. delicatula* egg masses from all storage period categories exposed to -40°C for 1 hour or 24 hours (**Figure 2**). Percentage parasitism was not affected by storage period on *L. delicatula* eggs in non-treated (i.e., the Unfrozen/parasitized treatment) (χ^2^ = 1.802, d.f. = 3, *p* = 0.614) and frozen at -40°C for 1 hour (χ^2^ = 2.979, d.f. = 3, *p* = 0.395). Significant differences were found in the percentage of parasitism between egg masses stored for different time periods when frozen at -40°C for 24 hours (χ^2^ = 12.235, d.f. = 3, *p* = 0.006) with significantly lower rates of parasitism being observed for eggs that were stored for eight months prior to freezing (*p* = 0.017) (**Figure 2**). The average parasitism rate for each treatment was 52.9 ± 4.9% (n = 47), 43 ± 4.4% (n = 43) and 29.4 ± 3.4% (n = 4 8) for the Unfrozen/parasitized, -40°C for 1 hour, and -40°C for 24 hours treatments, respectively. The parasitism rates for egg masses treated at -40°C for 24 hours were significantly lower than the parasitism rates obtained in the other two treatments (i.e., “Unfrozen/parasitized” and “1h.-40°C/parasitized” treatments) (χ^2^ = 12.221, d.f. = 2, *p* = 0.002) (**Figure 2**).

### Sex Ratio of Emerged *Anastatus orientalis* Offspring

**Figure 3:**
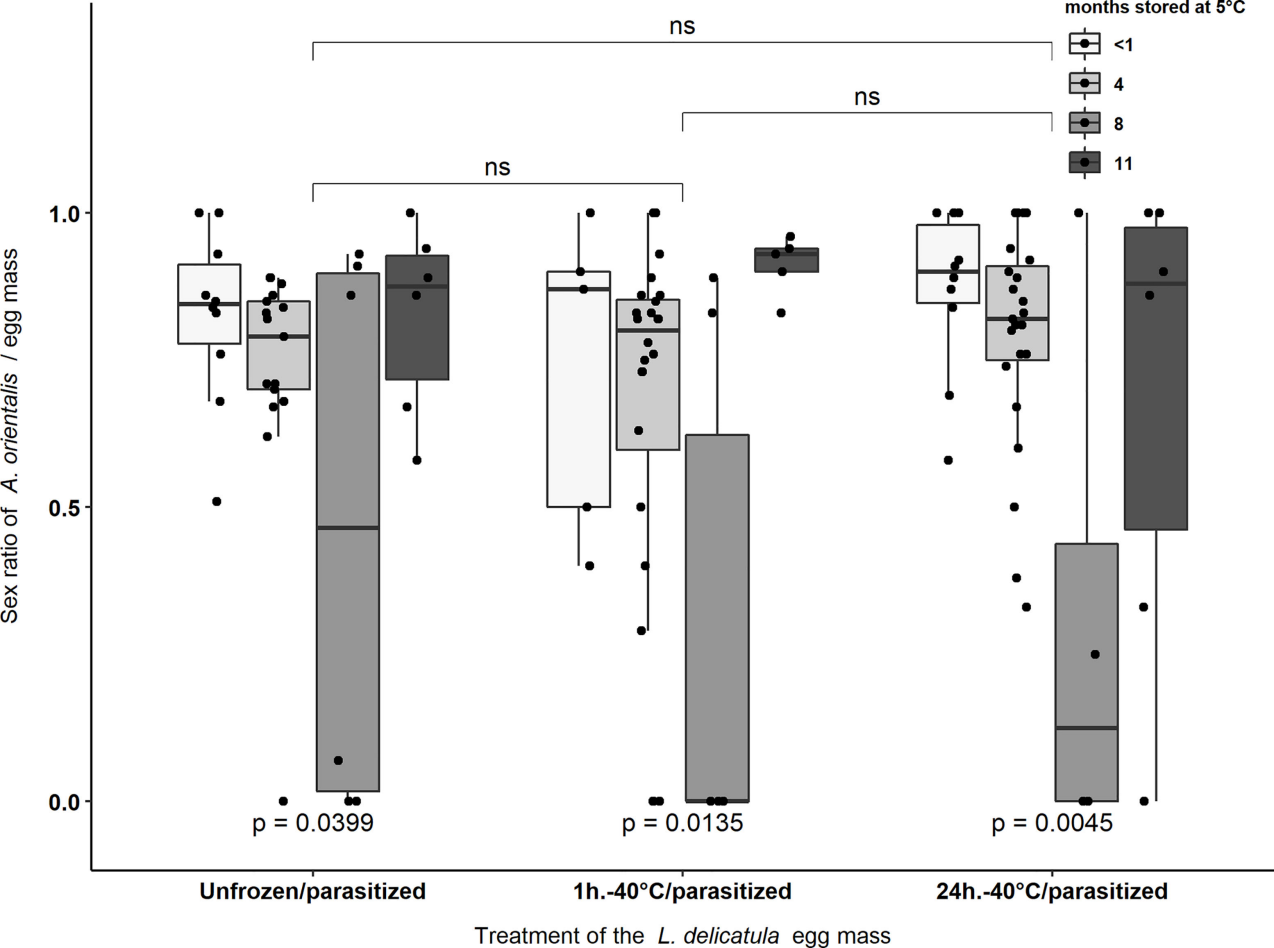
Sex ratio of *Anastatus orientalis* offspring that emerged from *L. delicatula* egg masses stored at <1, 4, 8, or 11 months at 5°C and treated at -40°C for 1 hour or 24 hours, or not exposed to -40°C. “ns” indicate non-significant differences in the sex ratio of emerged parasitoids between egg masses of different treatments. p-values < 0.05 indicate significant differences in the sex ratio of emerged parasitoids between egg masses stored for different periods (i.e., <1, 4, 8, or 11 months) within the same treatment group. Black dots represent data points.

For the total number of emerged parasitoids (n = 2,345) from all experimental egg masses (**Table 3**), 76.4% were females and 23.6% were males. An additional 21 live parasitoid larvae, pupae, or dead adults were found when dissecting *L. delicatula* eggs. These individuals were not included in sex ratio analyses. Sex ratio of emerged parasitoids was affected by the length of the storage period at 5°C (*F*_1,3_ = 9.72, *p* < 0.001) but was not affected by exposure to -40°C for 1 or 24 hours (*F*_1,2_ = 1.47, *p* = 0.23) or the interaction of cold storage period and freezing treatment (*F*_1,6_ = 0.93, *p* = 0.47). Egg masses stored for 8 months tended to have significantly male biased sex ratios compared to the sex ratios obtained from egg masses stored for <1, 4, or 11 months (**Figure 3**).

**Table 3:** Percentage of egg mass from which *Anastatus orientalis* emerged per treatment and cold storage period (n = total number of egg masses).

Time at -40°C	Name of the treatment	Cold storage periods at 5°C (months)			
		<1	4	8	11
1 hour	1h.-40°C.Parasitism	71.4% (n = 7)	100% (n = 24)	100% (n = 6)	83.3% (n = 6)
24 hours	24h.-40°C.Parasitism	90.9% (n = 11)	92% (n = 25)	66.7% (n = 6)	100% (n = 6)
No-treatment	Parasitism	66.7% (n = 15)	85% (n = 20)	100% (n = 6)	100% (n = 6)

### Hind Tibia Length of *Anastatus orientalis*

**Figure 4:**
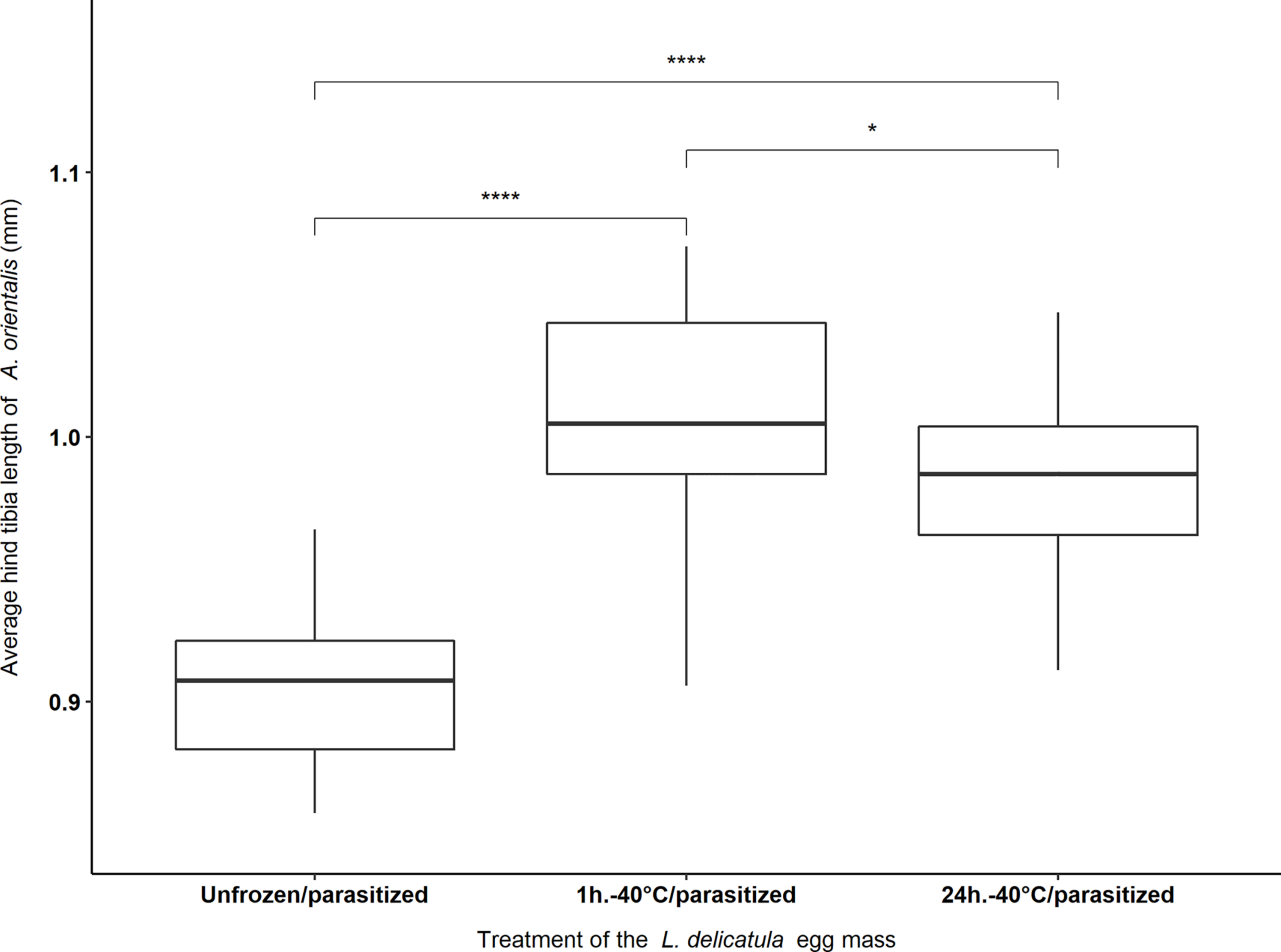
Average hind tibia length of female *Anastatus orientalis* offspring that emerged from *L. delicatula* egg masses stored for four months at 5°C and subjected to 1 hour or 24hours at -40°C, or no freezing (i.e., Unfrozen/parasitized). Asterisks indicate significant differences between different treatments [(*) = *p <*0.05; (****) = *p* < 0.001].

Significant differences existed between the right hind tibia length of female *A. orientalis* offspring among the three different treatments that were tested. This effect was significant for 4-month-old egg masses that were subjected to -40°C for 1 hour, and -40°C for 24 hours, and not exposed to -40°C (i.e., the Unfrozen/parasitized treatment) (F_2, 72_ = 48.65, *P* < 0.001). The average hind tibia length of female offspring was 1.005 ± 0.009 mm, 0.98 ± 0.007 mm and 0.907 ± 0.006 mm for “1h.-40°C/parasitized”, “24h.-40°C/parasitized” and the “Unfrozen/parasitized” treatments, respectively. The longest hind tibia, 1.072 mm, was measured for a female parasitoid that emerged from a 4-month-old egg mass from the “1h.-40°C/parasitized” treatment and the shortest hind tibia, 0.858 mm, was measured from a female parasitoid from the “Unfrozen/parasitized” treatment (**Figure 4**).

## Discussion

Exposing *L. delicatula* eggs to -40°C for either 1 or 24 hours completely prevented the development of *L. delicatula* nymphs in the 172 egg masses (i.e., 7,557 eggs) that were exposed to -40°C. This result indicates that the USDA-APHIS protocol used to kill *L. delicatula* nymphs in the UCR IQF, 72 hours at -40°C, could be reduced to a minimum of 1 hour and a maximum of 24 hours. Increased duration of storage periods at 5°C reduced the percentage of *L. delicatula* nymphs that emerged from both of the no-freeze treatments (i.e., Unfrozen and Unfrozen/parasitized treatments) from an average of ~73% of nymph emerged on egg masses stored at 5°C for <1 month (i.e., <30 days) to no nymphs emerging from egg masses stored for ~11 months (i.e., ~330 days). Previous studies have shown that storage periods up to 140 days at 5°C did not reduce the number of *L. delicatula* nymphs when compared to emergence rates from eggs that were <14 days of age at time of field collections, both with 100% of emergence (23). In this study, egg masses stored for a similar period of 120 days (i.e., 4 months) had a percentage of emergence of ~56%. The differences in percentage emergence of *L. delicatula* nymphs from egg masses with similar storage periods used in these two different studies could be due to varying time durations in the field prior to collection, variable shipping conditions during transit, especially during movement from field collection sites on the east coast of the U.S. (i.e., Pennsylvania) to the west coast quarantine facility in Riverside California, and subsequent differences in cold storage conditions in laboratories.

Exposure studies conducted here have demonstrated that *A. orientalis* can parasitize both frozen (i.e., -40°C treatments for 1 or 24 hours) *L. delicatula* egg masses and the respective non-frozen control treatments in the same storage period category (i.e., <1, 4, 8 and 11 months at 5°C). Parasitism rates in the non-frozen egg masses averaged ~50%, which is consistent with the ~40% parasitism rate found by Broadley et al. (18). The egg masses in both treatment groups, -40°C for 1 hour and 24 hours, exhibited maximum parasitism rates of 100% and 89% for each treatment, respectively, indicating that -40°C treated eggs were suitable for *A. orientalis* parasitism. Further, -40°C treated egg masses did not affect *A. orientalis* offspring sex ratios when compared with non-frozen controls. These two results indicate that egg masses treated at -40°C did not have a significant effect on the oviposition behavior of female *A. orientalis*. Additional studies are needed to confirm if -40°C treatments can increase the long-term cold storage options for use of *L. delicatula* egg masses for colony maintenance and experiments with egg parasitoids.

Other researchers have similarly demonstrated that *Anastatus* spp. are able to parasitize frozen host eggs. For example, Haye et al. (24), showed that *Anastatus bifasciatus* (Geoffroy) was able to parasitize both fresh and frozen eggs of *Halyomorpha halys* (Stål) (Hemiptera: Pentatomidae), an invasive agricultural pest. Additionally, Zhao et al. (25) demonstrated that *Anastatus fulloi* Sheng and Wang was able to parasitize (>80%) *Antheraea pernyi* (Guérin-Méneville) (Lepidoptera: Saturniidae) eggs that were stored at -5°C and -18°C for 6 to 12 months, respectively. Similarly, results reported here indicate that *A. orientalis* can parasitize *L. delicatula* egg masses that have been exposed to -40°C. Collectively, these examples suggest that frozen host eggs (i.e., Pentatomidae, Saturniidae and Fulgoridae) may not affect the acceptance behavior of foraging *Anastatus* spp. females. If this is correct, deployment of frozen *L. delicatula* egg masses could be used as sentinels to determine if the resident natural enemy fauna (e.g., parasitoids) in non-invaded areas are capable of successfully locating, parasitizing, and developing within *L. delicatula* eggs. Field studies of this kind may provide useful information on the levels of naturally occurring biotic resistance incipient *L. delicatula* populations could experience when invading new areas. Frozen sentinel egg masses could be deployed monthly during spring, summer and fall, for example, at study sites of interest to document levels of egg parasitoid activity and identities of species attacking frozen *L. delicatula* eggs.

The proportion of male *A. orientalis* that emerged from *L. delicatula* egg masses stored for 8 months at 5°C was significantly greater than that observed for egg masses stored for < 1 month, 4, or 11 months at 5°C. This result may have occurred because either the quality of *L. delicatula* eggs were affected by location and time of field collection or the nutritional value of the *L. delicatula* eggs stored for 8 months at 5°C was not optimal for *A. orientalis*. However, it seems unlikely that location and time of collection was a factor because egg masses used in all experiments reported on here were randomly selected from the same field locations and stored in the laboratory (5°C: 60% R.H.) under similar conditions. Sex allocation theory predicts the preferential placement of female eggs into higher quality host eggs and males into lower quality host eggs as a strategy to enhance the fitness of female offspring (26, 27). Accordingly, Zhao et al. (18) found that the percentage of female *A. fulloi* progeny decreased when ovipositing females were provided with *A. pernyi* eggs stored at -5°C to 3°C for 12 months when compared to fresh laid eggs. However, results reported here indicate that the female sex ratio of *A. orientalis* emerging from egg masses stored at 5°C for eleven months was not significantly different to the sex ratio of the egg masses stored for <1 month. Consequently, at this time, there appears to be no reasonable biological explanation as to why *L. delicatula* egg masses stored for eight months at 5°C in this study produced significantly more males especially when compared to egg masses that were stored for longer periods (i.e., 11 months at 5°C). Consequently, the low proportion of females that emerged from eggs stored at 5°C for eight months could be an artifact caused by males with low mating performance. If this assumption is correct, this could have resulted in female *A. orientalis* ovipositing fewer fertilized eggs when compared to females used in other treatments that mated with males with higher mating performance. Additionally, male parasitoids with low mating performance might also explain the low parasitism rates observed for egg masses stored for eight months, especially in the 24 hours at -40°C treatment, where significant differences in parasitism rates on egg masses from different storage periods were observed.

Hind tibia length, as a measure of body size, is used to estimate the fitness of parasitoids and the assumption is that larger individuals have longer tibia and are therefore likely to exhibit greater levels of fitness (26). Female *A. orientalis* that emerged from egg masses (i.e., cold stored at 5°C for 4 months) frozen for 1 hour at -40°C had significantly longer hind tibia lengths when compared to females that emerged from *L. delicatula* egg masses stored for four months that were either not frozen or frozen at -40°C for 24 hours. This result suggests that *L. delicatula* egg masses stored for four months at 5°C and then frozen at -40°C for 1 hour maybe more suitable for *A. orientalis* larval development than similarly aged egg masses that are stored at 5°C and not frozen or frozen at -40°C for 24 hours and then provided to ovipositing females. Similar studies on hemipteran (i.e., Pentatomidae) egg parasitoids (i.e. *Trissolcus* spp. [Hymenoptera: Platygastridae]) found that offspring that emerged from frozen (i.e., host eggs were held at -20°C or -80°C up to 4 years) host eggs had shorter hind tibia lengths when compare to offspring that emerged from non-frozen eggs (27, 28). Importantly, the positive effect of freezing four-month-old *L. delicatula* egg masses for 1 hour at -40°C on offspring size maybe the first time this effect has been demonstrated. However, further studies will be necessary to confirm and explain this potentially novel finding.

In conclusion, results presented here indicate that freezing *L. delicatula* egg masses of varying ages (i.e., eggs stored at 5°C for < 1 month, 4, 8, and 11 months) at -40°C for 1 or 24 hours results in 100% egg mortality, *A. orientalis* females can successfully parasitize eggs frozen at -40°C for 1 or 24 hours, and the fitness of offspring maybe enhanced if larvae develop in four month old egg masses that are frozen at -40°C for 1 hour. These results have significant practical applications. First this finding suggests that *L. delicatula* eggs exposed to -40°C for 1 to 24 hours is an effective and fast way to kill eggs making them amenable for safe removal from quarantine facilities. It may be possible to store egg masses at -40°C for considerable time periods without a loss in quality. If *L. delicatula* egg masses are not carefully managed, current storage practices (i.e., long-term storage at 5°C) can result in egg deterioration and mortality due to moisture related problems, especially the growth of saprophytic fungi (only egg masses that had no fungal contamination were used in these studies). It is possible that the quality, durability, and suitability of *L. delicatula* egg masses stored at -40°C for varying time periods for parasitism by *A. orientalis* could enhance long term storage options. However, this possibility needs experimental verification as storage at -40°C may inadvertently result in eggs of poor quality (e.g., desiccation) which could make them unusable. Finally, sentinel *L. delicatula* egg masses frozen at -40°C are killed which potentially allows for field deployment in non-invaded areas to proactively assess levels of parasitism and predation and possible identification of resident natural enemy species capable of attacking eggs in advance of an anticipated incursion by this pest.

## Data Availability Statement

The raw data supporting the conclusions of this article will be made available by the authors, without undue reservation.

## Author Contributions

FG-M conceived and designed the experiments, performed the experiments, analyzed the data, prepared figures and/or tables, authored or reviewed drafts of the paper, and approved the final draft. MH obtained the funding, authored or reviewed drafts of the paper, and approved the final draft. All authors contributed to the article and approved the submitted version.

## Funding

Funding for this work was provided by the California Department of Agriculture Office of Environmental Farming and Innovation’s Proactive Integrated Pest Management Solutions Grant Program, award number 18-0632-000-SG “Proactive Biological Control of Spotted Lantern Fly, Lycorma delicatula (Hemiptera: Fulgoridae)”.

## Conflict of Interest

The authors declare that the research was conducted in the absence of any commercial or financial relationships that could be construed as a potential conflict of interest.

## Publisher’s Note

All claims expressed in this article are solely those of the authors and do not necessarily represent those of their affiliated organizations, or those of the publisher, the editors and the reviewers. Any product that may be evaluated in this article, or claim that may be made by its manufacturer, is not guaranteed or endorsed by the publisher.
