# Supplementary material for: Effects of Freezing Lycorma delicatula Egg Masses on Nymph Emergence and Parasitization by Anastatus orientalis
Source: Front Insect Sci. 2022 Jul 12;2:937129. doi: 10.3389/finsc.2022.937129 (PMC10926522; doi:10.3389/finsc.2022.937129)
Supplement: Supplementary file 1 [file Table_1.docx]

**Supplementary Table 1.** Temperature by hours to simulate Beijing Fall climate conditions in temperature and humidity controlled cabinets.

| Time (24h) | | Temperature (°C) | |
| --- | --- | --- | --- |
| 0:00 | 17.92 | |  |
| 1:00 | 16.94 | |  |
| 2:00 | 16.19 | |  |
| 3:00 | 15.72 | |  |
| 4:00 | 14.00 | |  |
| 5:00 | 14.19 | |  |
| 6:00 | 14.74 | |  |
| 7:00 | 15.61 | |  |
| 8:00 | 16.75 | |  |
| 9:00 | 18.08 | |  |
| 10:00 | 19.50 | |  |
| 11:00 | 20.92 | |  |
| 12:00 | 22.25 | |  |
| 13:00 | 23.39 | |  |
| 14:00 | 24.26 | |  |
| 15:00 | 24.81 | |  |
| 16:00 | 25.00 | |  |
| 17:00 | 24.84 | |  |
| 18:00 | 24.37 | |  |
| 19:00 | 23.62 | |  |
| 20:00 | 22.64 | |  |
| 21:00 | 21.50 | |  |
| 22:00 | 20.28 | |  |
| 23:00 | 19.06 | |  |
